# Does sex shape the biological footprint of balloon-in-basket pulsed field ablation–guided pulmonary vein isolation? A biomarker analysis

**DOI:** 10.3389/fcvm.2026.1892008

**Published:** 2026-07-15

**Authors:** Jan-Per Wenzel, Kohei Ukita, Charlotte Eitel, Mirco Küchler, Karl-Heinz Kuck, Sascha Hatahet, Roland Richard Tilz

**Affiliations:** Department of Rhythmology, University of Lübeck, Lübeck, Germany

**Keywords:** atrial fibrillation, biomarker, catheter ablation, pulmonary vein isolation, pulsed field ablation

## Abstract

**Background:**

The balloon-in-basket pulsed field ablation (BiB-PFA) system is a novel modality that achieves myocardial-selective ablation with minimal collateral tissue injury. Sex-related differences in biomarker responses following BiB-PFA-guided pulmonary vein isolation (PVI) for atrial fibrillation (AF) remain unclear.

**Objective:**

We aimed to investigate sex-based differences in peri-procedural biomarker responses and procedure-related complications among patients undergoing PVI alone using the BiB-PFA system.

**Methods:**

We retrospectively analyzed patients undergoing initial PVI with the BiB-PFA system between January 2024 and March 2026. Peri-procedural inflammatory, myocardial injury, hemolysis, and renal biomarkers were assessed pre-ablation and on the first post-procedural day. Sex-based differences in biomarker changes (Δ = post – pre) and procedural safety outcomes were compared.

**Results:**

A total of 50 patients were included (50% female). Baseline characteristics and procedural parameters were comparable between groups. Δ values in inflammatory markers (Δwhite blood cell count: *P* = 0.813; ΔC-reactive protein: *P* = 0.343), myocardial injury markers (Δcreatine kinase: *P* = 0.261; Δtroponin T: *P* = 0.078), hemolysis markers and renal function parameters (Δtotal bilirubin: *P* = 0.160; Δhaptoglobin: *P* = 0.104; Δestimated glomerular filtration rate: *P* = 0.984; Δmyoglobin: *P* = 0.258) did not differ significantly between female and male patients. Procedure-related complications were rare and showed no sex-related differences.

**Conclusions:**

No statistically significant sex-related differences were detected in peri-procedural biomarker responses or observed procedural complications following BiB-PFA-guided PVI.

## 1 Introduction

Pulsed field ablation (PFA), a novel ablation modality based on irreversible electroporation, has gained increasing attention due to its myocardial selectivity and reduced risk of collateral tissue injury (1).

The balloon-in-basket (BiB)-PFA is a novel system that integrates a compliant balloon with an expandable multielectrode lattice structure, designed to achieve stable catheter positioning and consistent electrode-tissue contact (2–4). Several studies have focused on the periprocedural inflammatory, myocardial injury, and hemolysis markers associated with pulmonary vein isolation (PVI) using the BiB-PFA system in patients with atrial fibrillation (AF) (5–8). However, sex-related differences in post-procedural biomarker responses following the BiB-PFA-guided PVI remain poorly characterized.

In this report, we aimed to investigate sex-based differences in peri-procedural inflammatory, myocardial injury, and hemolysis markers among patients undergoing PVI using the BiB-PFA system.

## 2 Materials and methods

### 2.1 Study design and population

This study included patients who underwent PVI alone using the BiB-PFA system (VOLT™, Abbott) as an initial catheter ablation for AF between January 2024 and March 2026 at the Heart Center Lübeck, and who had complete periprocedural biomarker data available. Patient characteristics, procedural details, and differences in biomarkers measured during the procedure and on the following day were compared between female and male patients. The study protocol was approved by the local ethics committee (Lübeck Ablation Registry, approval number WF-028/15) and conducted in accordance with the Declaration of Helsinki. Written informed consent for catheter ablation and participation in the study was obtained from all patients.

### 2.2 Ablation procedure

All patients underwent a standardized preprocedural work-up in line with institutional protocols (9). Ablation procedures were performed under either deep sedation with propofol or light sedation without propofol (midazolam, fentanyl, metamizole, and lidocaine) at the discretion of the treating electrophysiologist. Vascular access was obtained via two ultrasound-guided punctures of the femoral vein using 8 Fr sheaths. A diagnostic catheter was placed in the coronary sinus. Transseptal puncture was performed with a modified Brockenbrough technique under fluoroscopic guidance, and unfractionated heparin was administered to maintain an activated clotting time >300 s. A steerable 13 Fr sheath (Agilis™ NxT, Abbott) was used to introduce the BiB-PFA catheter into the left atrium (LA). The three-dimensional geometry of the LA and PVs was reconstructed using a mapping system (EnSite™ X, Abbott). PVI was performed with at least two rotated applications per PV at nominal voltage (1,800 V). If phrenic nerve capture was detected in right-sided PVs, energy delivery was reduced to 1,400 V with a minimum of three applications. A maximum of eight applications per PV was permitted. Acute isolation was confirmed by remapping with the BiB-PFA catheter.

### 2.3 Postprocedural management

Hemostasis was achieved with vascular closure devices or figure-of-eight sutures combined with compression bandage. The bandage was removed at least one hour after the end of the ablation procedure, and the suture was removed four hours after the bandage removal. Transthoracic echocardiography was performed immediately after the procedure, at one hour, and on the first postprocedural day to exclude pericardial effusion.

### 2.4 Blood sampling and analysis

Venous blood samples were obtained at two time points: (1) after femoral venous access prior to ablation, and (2) on the morning of the first post-procedural day (5–8). Analyzed biomarkers included white blood cell (WBC) counts and C-reactive protein (CRP) as inflammatory markers, creatine kinase (CK) and troponin T as myocardial injury markers, and hemoglobin, total bilirubin, haptoglobin, estimated glomerular filtration rate (eGFR), and myoglobin as hemolysis-related markers as well as renal function parameters. All samples were obtained under fasting conditions and analyses were performed in the central clinical chemistry laboratory of the University Hospital Schleswig-Holstein according to standardized protocols. eGFR was calculated using the CKD-EPI 2021 equation.

### 2.5 Statistical analysis

Categorical variables were expressed as counts and percentages. Given the small sample size, all continuous variables were expressed as median (interquartile range), and non-parametric methods were used for all group comparisons. Between-group comparisons were performed using the Mann–Whitney *U* test, and within-group comparisons using the Wilcoxon signed-rank test. Categorical variables were compared using Fisher's exact test or chi-squared test, as appropriate. Correlations between biomarker changes and PFA applications were analyzed using Pearson or Spearman coefficients, depending on data distribution. A *p* value < 0.05 was considered statistically significant. All statistical analyses were performed using JMP 19 software (SAS Institute).

## 3 Results

### 3.1 Patient characteristics

**Figure 1:**
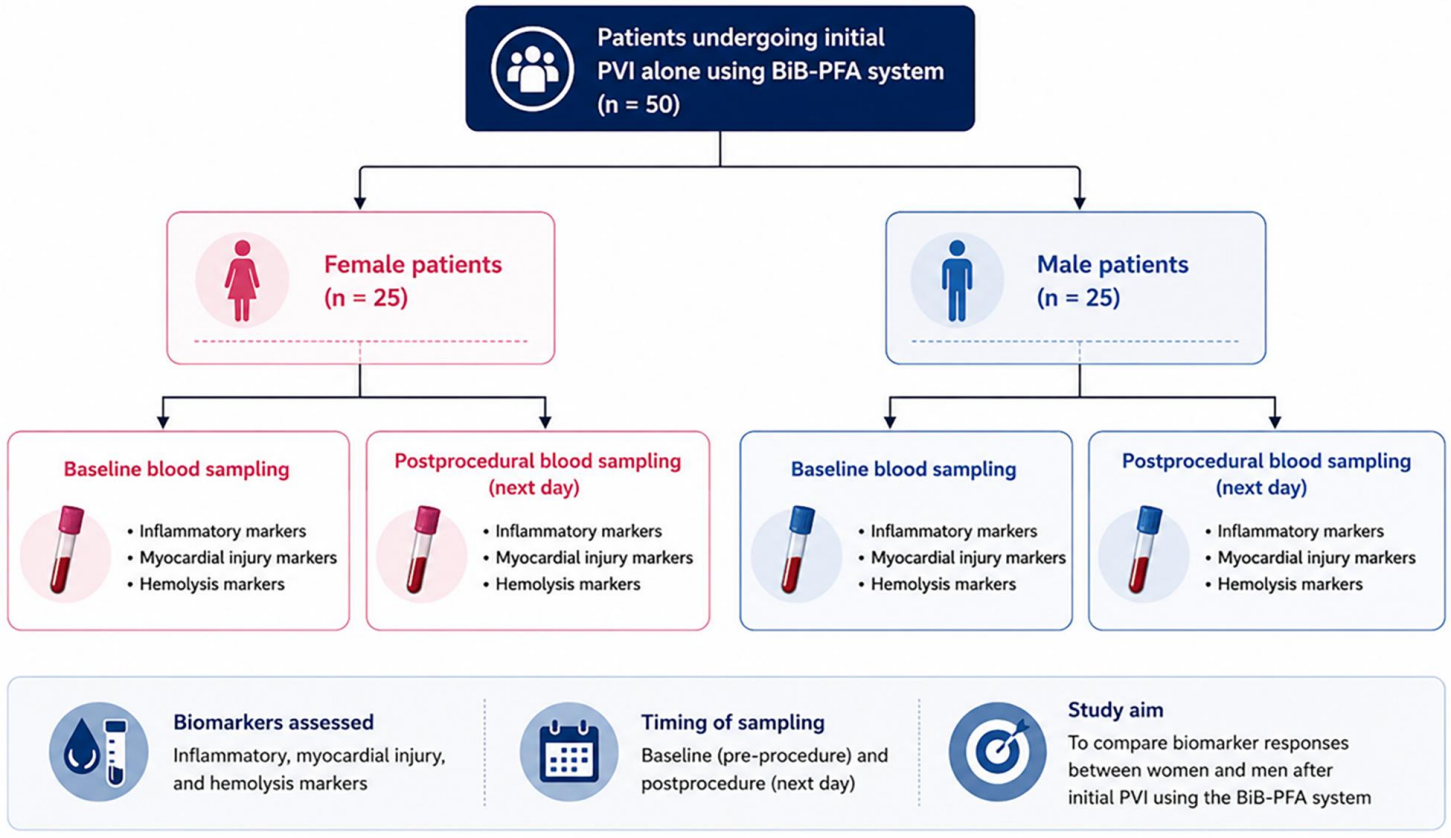
Study flow chart. A total of 50 patients were included.

A total of 50 patients were included (Figure 1). Baseline characteristics are summarized in Table 1. Of 50 patients, 25 were female. There were no significant differences in age, body mass index, AF type, incidence of hypertension, diabetes mellitus, and coronary artery disease, CHA_2_DS_2_-VA score, left ventricular ejection fraction (LVEF), and left atrial volume index (LAVI) between female and male patients.

**Table 1:** Baseline characteristics of the patients.

Variable	Female (*n* = 25)	Male (*n* = 25)	*P* value
Age (years)	65 (62, 77)	64 (57, 71)	0.115
Body mass index (kg/m^2^)	26 (22, 32)	27 (25, 30)	0.871
AF type			
Paroxysmal AF, *n* (%)	15 (60)	13 (52)	0.569
Non-paroxysmal AF, *n* (%)	10 (40)	12 (48)	0.569
Hypertension, *n* (%)	14 (56)	14 (56)	1.000
Diabetes mellitus, *n* (%)	4 (16)	4 (16)	1.000
Coronary artery disease, *n* (%)	5 (20)	3 (12)	0.440
CHA_2_DS_2_-VA score	2 (1, 3)	1 (1, 3)	0.134
NT-proBNP (ng/L)	503 (176, 1,338)	213 (55, 1,397)	0.217
LVEF (%)	55 (55, 60)	55 (55, 60)	0.910
LAVI (mL/m^2^)	36 (33, 45)	31 (28, 47)	0.375

AF, atrial fibrillation; LAVI, left atrial volume index; LVEF, left ventricular ejection fraction; NT-proBNP, N-terminal pro-brain natriuretic peptide.

### 3.2 Procedural details and complications

Procedural details and procedure-related complications are shown in Table 2. Successful PVI was achieved in all patients. There were no significant differences in procedure time, dose area product, number of total applications, contrast amount, and the use of vascular closure devices between female and male patients. The incidence of procedure-related complications including cardiac tamponade, stroke, phrenic nerve injury, femoral pseudoaneurysm, and atrioesophageal fistula was low and did not differ between sexes.

**Table 2:** Procedural details and complications.

Variable	Female (*n* = 25)	Male (*n* = 25)	*P* value
Procedural details			
Deep sedation with propofol, *n* (%)	18 (72)	14 (56)	0.239
Successful PVI, *n* (%)	25 (100)	25 (100)	1.000
Procedure time (min)	66 (55, 73)	53 (34, 71)	0.057
Left atrial dwell time (min)	52 (46, 59)	42 (35, 48)	0.101
Fluoroscopy time (min)	9 (7, 13)	6 (5, 10)	0.060
Dose area product (cGy*cm^2^)	309 (286, 475)	390 (287, 583)	0.311
Number of total applications	16 (13, 17)	16 (13, 16)	0.771
Number of applications for LSPV	4 (4, 4)	4 (3, 4)	
Number of applications for LIPV	4 (4, 4)	4 (3, 4)	
Number of applications for RSPV	4 (3, 4)	4 (3, 4)	
Number of applications for RIPV	3 (3, 4)	4 (3, 4)	
Contrast amount (mL)	45 (40, 50)	40 (40, 50)	0.758
Use of vascular closure devices, *n* (%)	9 (36)	7 (28)	0.544
Procedure-related complications			
Cardiac tamponade, *n*	0	0	1.000
Stroke, *n*	0	0	1.000
Phrenic nerve injury, *n*	0	0	1.000
Femoral pseudoaneurysm, *n*	1	0	0.312
Atrioesophageal fistula, *n*	0	0	1.000

LIPV, left inferior pulmonary vein; LSPV, left superior pulmonary vein; PVI, pulmonary vein isolation; RIPV, right inferior pulmonary vein; RSPV, right superior pulmonary vein.

### 3.3 Pre- and post-ablation biomarkers

**Figure 2:**
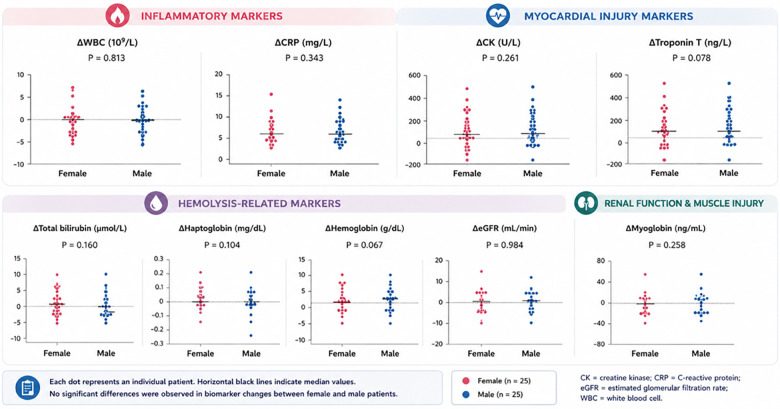
Intergroup comparisons of delta values. Changes in biomarkers did not differ significantly between female and male. CK, creatine kinase; CRP, C-reactive protein; eGFR, estimated glomerular filtration rate; WBC, white blood cell.

Inflammatory, myocardial injury, and hemolysis-related markers and renal function before and after ablation are shown in Table 3. At baseline, there were no significant differences between female and male patients in any of the biomarkers. Intergroup comparisons of delta values (Δ = post – pre) are shown in Figure 2. Changes in WBC, CRP, CK, total bilirubin, haptoglobin, eGFR, and myoglobin did not differ significantly between female and male patients. In addition, female patients demonstrated numerically greater increases in troponin T concentrations than male patients; however, this difference did not reach statistical significance (*P* = 0.078).

**Table 3:** Pre- and post-ablation biomarkers.

Parameter	Timing	Female (*n* = 25)	Male (*n* = 25)	*P* value
Inflammatory markers				
WBC (10^9^/L)	Pre	7.1 (5.2, 8.5)	5.9 (4.9, 7.6)	0.078
	Post	8.3 (7.1, 9.1)	7.5 (6.3, 9.1)	0.502
CRP (mg/L)	Pre	1.4 (1.0, 2.3)	1.0 (0.6, 1.5)	0.181
	Post	5.7 (3.4, 7.4)	5.3 (3.2, 7.3)	0.973
Myocardial injury markers				
CK (U/L)	Pre	86 (66, 122)	81 (69, 112)	0.506
	Post	345 (237, 473)	294 (206, 393)	0.260
Troponin T (ng/L)	Pre	7.4 (5.3, 10.6)	8.1 (5.7, 11.8)	0.803
	Post	1,065 (867, 1,425)	1,000 (707, 1,189)	0.834
Hemolysis markers				
Hemoglobin (g/dL)	Pre	13.5 (12.8, 14.2)	14.1 (13.1, 15.0)	0.275
	Post	12.7 (12.4, 13.3)	13.8 (12.7, 14.6)	0.067
Total bilirubin (µmol/L)	Pre	8.3 (6.9, 11.4)	10.0 (8.1, 12.7)	0.054
	Post	10.3 (6.8, 13.6)	11.3 (8.4, 16.8)	0.119
Haptoglobin (mg/dL)	Pre	1.1 (0.7, 1.6)	1.0 (0.7, 1.3)	0.276
	Post	1.5 (0.8, 1.8)	1.0 (0.7, 1.4)	0.075
eGFR (mL/min/1.73 m^2^)	Pre	79 (65, 86)	85 (67, 95)	0.091
	Post	75 (66, 86)	82 (68, 94)	0.057
Myoglobin (ng/mL)	Pre	38 (29, 50)	41 (35, 50)	0.268
	Post	51 (48, 74)	48 (41, 68)	0.304

CK, creatine kinase; CRP, C-reactive protein; eGFR, estimated glomerular filtration rate; WBC, white blood cell.

### 3.4 Correlation between energy applications and biomarker changes

As shown in Table 4, no correlation was observed between the number of energy deliveries and changes in any of the evaluated biomarkers.

**Table 4:** Correlation between energy applications and biomarker changes.

Marker	Female (*n* = 25)	*P* value	Male (*n* = 25)	*P* value
WBC	0.073	0.725	0.172	0.414
CRP	0.012	0.954	0.126	0.547
CK	0.179	0.391	0.211	0.312
Troponin T	0.311	0.129	0.222	0.293
Hemoglobin	0.079	0.713	0.064	0.762
Total bilirubin	0.146	0.492	0.132	0.531
Haptoglobin	0.174	0.410	0.065	0.759
eGFR	−0.018	0.935	−0.164	0.431

CK, creatine kinase; CRP, C-reactive protein; eGFR, estimated glomerular filtration rate; WBC, white blood cell.

## 4 Discussion

### 4.1 Main findings

This is the first study to compare biomarker responses after the BiB-PFA-guided PVI between sexes. Our main findings are: no statistically significant sex-related differences were detected in peri-procedural biomarker responses or observed procedural complications following BiB-PFA-guided PVI.

### 4.2 Comparison with previous studies

Prior studies have reported sex-related differences in outcomes after AF ablation using conventional thermal energy sources such as radiofrequency or cryoballoon, with women experiencing higher complication rates and more pronounced inflammatory responses, potentially due to differences in tissue characteristics or hormonal influences (10–12). However, the advent of PFA, with its tissue-selective mechanism, may attenuate these differences. Previous investigations of the BiB-PFA system have demonstrated relatively modest increases in inflammatory and myocardial injury markers compared with thermal ablation modalities, supporting the concept of reduced collateral damage (5–8). Our findings extend these observations by showing that such favorable biomarker profiles are consistent across sexes. One possible explanation may be the tissue-selective mechanism of irreversible electroporation; however, the present study was not designed to directly investigate mechanistic pathways, and this interpretation should therefore be considered hypothesis-generating.

### 4.3 Sex-related differences in biomarker responses

Several mechanisms may explain the absence of sex-related differences observed in this study. First, PFA induces cell death through disruption of cell membrane integrity rather than thermal injury, thereby minimizing variability related to tissue thickness or blood flow, factors that may differ between women and men. Second, the balloon-in-basket design may provide more uniform electrode–tissue contact and energy delivery, reducing operator- and anatomy-dependent variability. Third, the standardized procedural workflow and comparable procedural characteristics between groups likely contributed to the uniform biological response.

Although no statistically significant sex-related differences were detected, female patients exhibited a numerically greater increase in troponin T concentrations compared with male patients (*P* = 0.078). Given the limited sample size and single post-procedural sampling time point, this observation should be interpreted cautiously and warrants confirmation in larger prospective studies.

### 4.4 Clinical implications

The present findings have important clinical implications. Given that women have historically been underrepresented in AF ablation studies and may have experienced less favorable outcomes with conventional techniques, the demonstration of comparable biological responses with BiB-PFA is reassuring. These results support the notion that PFA-based technologies may help reduce sex disparities in AF ablation by offering a more reproducible and tissue-selective approach. Furthermore, the lack of significant hemolysis or renal impairment differences between sexes suggests that the safety profile of BiB-PFA is consistent regardless of patient sex, which is particularly relevant in populations at higher risk for procedural complications.

### 4.5 Limitations

Several limitations should be acknowledged. First, this was a single-center study with a relatively small sample size, which may limit statistical power to detect subtle sex-related differences. Importantly, the study was not powered to formally demonstrate equivalence between female and male patients, and therefore the absence of statistically significant differences should not be interpreted as proof of biological equivalence. Second, biomarkers were obtained only once after the procedure, on the morning of the first post-procedural day. Consequently, temporal biomarker kinetics, including onset, peak response, and recovery phases, could not be evaluated. In addition, exact intervals between procedure completion and post-procedural blood sampling were not prospectively recorded. This may have introduced variability, particularly for biomarkers with time-dependent release patterns such as troponin T, bilirubin, haptoglobin, and myoglobin. Third, although procedural characteristics were similar between groups, unmeasured confounders may have influenced the results. Owing to the limited sample size, multivariable analyses were not performed because reliable adjustment for multiple covariates would likely result in model overfitting. Furthermore, sedation strategy may influence inflammatory biomarker responses. Although sedation modalities were similarly distributed between groups, a residual confounding effect cannot be completely excluded. Fourth, the number of energy applications may not fully reflect effective tissue energy exposure. Factors such as catheter–tissue contact, anatomical variability, vein-specific application patterns, and procedural complexity may influence biological responses and are not captured by application count alone. Fifth, long-term outcomes, including arrhythmia recurrence, repeat ablation procedures, symptom burden, and quality-of-life measures, were not evaluated and therefore the clinical implications of the observed biomarker responses remain uncertain. Sixth, the study focused exclusively on initial PVI without additional substrate modification, and therefore the findings may not be generalizable to more complex ablation strategies. Finally, no correction for multiple comparisons was applied; therefore, *p*-values should be interpreted as exploratory.

## 5 Conclusions

In patients undergoing initial PVI using the BiB-PFA system, no statistically significant sex-related differences were detected in peri-procedural inflammatory, myocardial injury, hemolysis-related, or renal biomarker responses. Likewise, no sex-related differences were observed in procedural complications. Given the limited sample size, these findings should be considered hypothesis-generating and require confirmation in larger prospective studies.

## Data Availability

The raw data supporting the conclusions of this article will be made available by the authors, without undue reservation.
